# Screening and Identification of *Latilactobacillus curvatus Z12* From Rumen Fluid of an Adult Female Sika Deer as a Potential Probiotic for Feed Additives

**DOI:** 10.3389/fvets.2021.753527

**Published:** 2021-10-21

**Authors:** Yan Zhang, Shuang Liang, Meng Di Zhao, Xue Yang, Seong Ho Choi, Guang Yu Li

**Affiliations:** ^1^College of Animal Science and Technology, Qingdao Agricultural University, Qingdao, China; ^2^Department of Animal Science, Chungbuk National University, Cheongju, South Korea; ^3^Department of Animal Science, College of Animal Sciences, Jilin University, Changchun, China; ^4^Department of Special Economic Animal Nutrition and Feed Science, Institute of Special Animal and Plant Sciences, Chinese Academy of Agricultural Sciences, Changchun, China

**Keywords:** rumen, lactic acid bacteria, probiotic, antimicrobial activity, sika deer health

## Abstract

Lactic acid bacteria (LAB) are the main microorganisms used as probiotics against gastrointestinal inflammation. The objective of this study was to evaluate the potential probiotic characteristics (antimicrobial activity, artificial gastrointestinal model resistance, cell surface hydrophobicity, and autoaggregation ability) and safety characteristics (hemolytic activity, antimicrobial resistance, and *in vivo* safety) of LAB isolated from the rumen fluid of an adult female sika deer. Two isolated strains identified as *Latilactobacillus curvatus Z12* and *Z19* showed good antimicrobial activity against enteropathogenic *Escherichia coli* (ATCC25922), *Salmonella typhi* (ATCC14028), and *Staphylococcus aureus* (ATCC25923). In addition, *L. curvatus Z12* exhibited higher artificial gastrointestinal model resistance, cell surface hydrophobicity and autoaggregation ability than *L. curvatus Z19*. Therefore, regarding safety characteristics, only *L. curvatus Z12* was evaluated. Upon assessment of safety, *L. curvatus Z12* was negative for hemolytic activity and susceptible to penicillin G and cefamandole. Furthermore, an *in vivo* safety assessment showed that high-dose *L. curvatus Z12* (10^9^ CFU/mL) supplementation not only had no adverse effects on body weight gain, feed intake, and organ coefficients of treated mice but also played a key role in promoting the immune system maturation of treated mice. This research revealed that *L. curvatus Z12* possesses desirable probiotic characteristics and could be used as a potential probiotic feed additive to improve sika deer health.

## Introduction

Sika deer (*Cervus nippon*) is an important economic animal in China due to its velvet antlers, which are used in traditional Chinese medicine (1, 2). With overhunting and habitat fragmentation, the wild sika deer population has been markedly reduced, and captivity breeding of sika deer has become the main method to reduce the pressure on wild sika deer resource depletion. However, diarrhea has become a common disease in captive-bred young sika deer, and severe cases can result in death, leading to significant economic losses. Therefore, diarrhea has turned into a focus of disease control and prevention for captivity breeding of sika deer, although related studies remain lacking.

The gastrointestinal tract (GIT) of ruminants contains a complex and diverse population of microorganisms that provide many beneficial functions to the host. Many studies have reported that the composition and dynamics of the intestinal microbiota are closely related to the occurrence of intestinal diseases (3–5). Once the intestinal microbiota is invaded by exogenous pathogens, the balance of the gut barrier is destroyed, leading to digestive system pathologies such as infectious diseases, metabolic disorders, and intestinal diseases (6). Historically, antibiotics have played an important role in animal husbandry and have been used as feed additives to improve gastrointestinal disorders (7, 8). However, in recent years, the use of antibiotics in animals has been gradually banned due to antibiotic residues in food and increasing bacterial resistance (9). In addition, a recent study showed that the use of therapeutic antibiotics negatively affects the temporal development of GIT microbiota diversity and stability in calves (10). Therefore, finding antibiotic alternatives is very important for livestock animals. Probiotics are living microorganisms that, given in sufficient amounts, have the potential to improve the health of host animals (11). Many studies have reported that probiotic supplementation can not only decrease the incidence of intestinal infections in many animals but also improve growth performance and feed efficiency (12–14). Lactic acid bacteria (LAB) are the main representatives of probiotics and have a long history of use as probiotics (15). They have many benefits for host health, such as promoting the development of the host GIT, enhancing the immune system, and alleviating inflammatory bowel diseases (16, 17). Furthermore, LAB can produce various metabolites, such as hydrogen peroxide, bacteriocins and organic acids (lactic acid and propionic acid), which play a key role in regulating the balance of intestinal microorganisms (18). Therefore, LAB as substitutes for antibiotics have attracted extensive attention in the treatment of animal gastrointestinal diseases (19). One study has shown that LAB can improve the daily gain and diarrhea incidence of dairy calves (20). Studies have also found similar results in other animals, such as poultry (12), silver foxes, and raccoon dogs (21). Therefore, in recent years, there has been increasing interest in the isolation of new probiotics that produce antimicrobial substances, which will become an ongoing practice (22).

LAB isolated from their natural hosts are preferred because they are better adapted to the environment of the GIT, are able to spontaneously proliferate, and have better therapeutic and health care effects than strains isolated from other sources (23). In the present study, some probiotic characteristics (antimicrobial activity, survival in artificial gastrointestinal models, cell surface hydrophobicity (CSH), and autoaggregation ability) and safety characteristics (hemolytic activity, antimicrobial resistance, and *in vivo* safety) of *L. curvatus* isolated from fresh rumen fluid of an adult domestic female sika deer were evaluated.

## Materials and Methods

### Sampling

A 50 mL ruminal fluid sample was collected via oral tubing (24) from the rumen of a healthy domestic adult female sika deer that had not received antibacterial treatment for more than half a year and was housed at the research farm center of the Institute of Special Animal and Plant Sciences, Chinese Academy of Agricultural Sciences, in Zuojia, Jilin, China, before morning feeding. The ruminal fluid sample was filtered with four layers of sterile cheesecloth to remove large particles of feed, flushed with CO_2_, and quickly transported on ice to our laboratory for further analysis.

### Isolation and Screening of LAB

The rumen fluid sample was diluted from 10^−1^ to 10^−6^ using PBS by the 10-fold dilution method. An aliquot of 100 μL of each dilution was pipetted in triplicate onto Man, Rogosa and Sharpe (MRS) agar plates with CaCO_3_ containing peptone (10.0 g/L), beef extract (10.0 g/L), glucose (20.0 g/L), yeast extract (5.0 g/L), sodium acetate (2.0 g/L), ammonium citrate dibasic (2.0 g/L), K_2_HPO_4_ (1.0 g/L), MnSO_4_ (1.0 g/L), MgSO_4_.7H_2_O (0.2 g/L), Tween 80 (1 mL/L), agar (20.0 g/L), and CaCO_3_ (20.0 g/L) and spread with a glass spreader. After 48 h of anaerobic inoculation at 37°C, the single colony with the maximum clearing zone of CaCO_3_ disappearance was selected for further screening and Gram staining. Finally, purified and isolated single colonies were stored in MRS broth (without agar) with sterile 20% (v/v) glycerol (Sigma, USA) solution at −80°C.

### Antimicrobial Activity

The antimicrobial activity of the isolated strains was determined by a well-diffusion assay (25, 26). Briefly, the isolated bacterial strains were cultured overnight in MRS broth at 37°C (10^9^ CFU/mL), and the bacterial suspension (BS) was centrifuged at 4°C and 8,000 rpm for 10 min. The cell-free supernatant (CFS) was removed, and the bacterial pellet (BP) was resuspended in the same volume of PBS. Sterile metal cylinders were placed on Luria–Bertani (LB) agar plates containing peptone (10.0 g/L), beef extract (1.0 g/L), yeast extract (5.0 g/L), NaCl (5.0 g/L), and agar (20.0 g/L) at a final concentration of 10^6^ CFU/mL pathogenic indicator bacteria (enteropathogenic *Escherichia coli*, ATCC25922; *Salmonella typhi*, ATCC14028; *Staphylococcus aureus*, ATCC25923) to prepare the wells, and each well was inoculated with 100 μL of MRS broth and the BS, CFS, BP, and CFS _pH7.0_ of the isolated strains. Eventually, the LB agar plates were incubated at 37°C for 24 h, and the indicator bacterial growth inhibition zone was measured with a Vernier caliper. Separate experiments were performed in triplicate.

### Morphological Identification of LAB

The morphological characteristics of isolated strains were observed and recorded under an optical microscope (Leica, Germany) before and after Gram staining to preliminarily identify the species of the isolated strains.

### Molecular Identification of LAB

Total genomic DNA was extracted using a DNA extraction kit (Tiangen, China) according to the manufacturer's protocol. Bacterial 16S rDNA amplification was carried out by polymerase chain reaction (PCR) using the universal primers 16S rDNA-F (5′- AGAGTTTGATCMTGGCTCAG−3′) and 16S rDNA-R (5′- GGTTACCTTGTTACGACTT−3′). The PCR parameters were as follows: denaturation at 95°C for 2 min, followed by 30 cycles of 94°C for 20 s, 60°C for 20 s, and 72°C for 40 s, and a final extension at 72°C for 1 min. After the reaction, the PCR amplification products were identified by 1% agarose gel electrophoresis and purified by a DNA gel extraction kit (Thermo, USA). The purified PCR products were sent to Shanghai Biotechnology Engineering Co. Ltd., for identification, the sequence files were input into the NCBI BLAST program for homology comparison to obtain information about the strains with the sequences most similar to that of the isolated bacteria, and then, a phylogenetic tree was constructed via MEGA 7.0 software.

### Resistance to Artificial Gastrointestinal Models

Artificial gastrointestinal models simulating the stomach and intestine were prepared according to a previous method (27) (rumen simulation was not considered, mainly because the isolates were derived from rumen fluid). Bacterial pellets from MRS broth cultures at a concentration of approximately 10^9^ CFU/mL were resuspended in artificial gastric juice (pH 2.0) and incubated for 1.5 h at 37°C with stirring at 200 rpm. After incubation, the bacterial pellets were washed twice with PBS by centrifugation at 8,000 rpm and 4°C for 10 min and then resuspended in artificial intestinal fluid and incubated for 2 h at 37°C with stirring at 200 rpm. Bacterial pellet viability was quantified by the dilution method of plate counting before and after incubation in artificial gastric juice and intestinal fluid. Separate experiments were performed in triplicate. The bacterial survival rate was calculated by the following formula:


(1) $$\begin{array}{r} \text{Survival rate }\left(\%\right)=T_{\text{assay}}/T_{\text{initial}}\times \;100\% \end{array}$$

where T_initial_ and T_assay_ are the number of viable bacteria in pellets (log CFU/mL) before incubation in the artificial gastric juice or intestinal fluid and after incubation in the artificial gastric juice or intestinal fluid, respectively.

### Cell Surface Hydrophobicity

The CSH of bacteria was determined by spectrophotometry. The isolated strains were cultured in MRS broth at 37°C for 18–24 h and then centrifuged at 8,000 rpm and 4°C for 10 min. The resulting bacterial pellets were washed two times with PBS and adjusted to 10^8^ CFU/mL (OD_630nm_ = 0.11 ± 0.005; A_1_). Then, xylene and chloroform were added at a ratio of 1:1, followed by vortexing for 3 min and then incubating at 37°C for 3 h. Finally, the aqueous phase was carefully absorbed, and the OD_630nm_ (A_2_) was measured. Separate experiments were performed in triplicate. The percentage of CSH was calculated by the following formula:


(2) $$\begin{array}{r} \text{CSH }\left(\%\right)=\left(1-A_{2}/A_{1}\right)\;\times 100\% \end{array}$$

where A_1_ and A_2_ are the absorbances before and after incubation for 3 h, respectively.

### Autoaggregation Ability

The method of Wang et al. (28) was used to determine autoaggregation, with some modifications. In brief, the isolated strains were cultured in MRS broth at 37°C for 18–24 h (10^9^ CFU/mL). The pellets were harvested by centrifugation at 8,000 rpm and 4°C for 10 min, washed two times with PBS, and resuspended in the same volume of PBS to record the OD_630nm_ (A_3_). Then, the bacterial solution was vortexed for 10 s and incubated at 37°C. After 8 h, the upper part was carefully absorbed, and the absorbance was measured at 630 nm (A_4_). Separate experiments were performed in triplicate. The autoaggregation ability rate was calculated by the following formula:


(3) $$\begin{array}{r} \text{Autoaggregation ability }\left(\%\right)=\left(1-A_{4}/A_{3}\right)\;\times 100\% \end{array}$$

where A_3_ and A_4_ are the absorbances before and after incubation for 8 h, respectively.

### Safety Assessment of LAB

#### Hemolytic Activity

Determination of hemolytic activity followed the method described by Reuben et al. (23). After culturing overnight in MRS broth, the isolated strain was streaked on sheep blood agar plates (Oxoid, Germany) and incubated at 37°C for 48 h. Blood lysis zones that appeared around the bacterial colonies were considered to indicate that the strains were hemolytic (β-hemolysis), while green-hued zones (α-hemolysis) or lack of any zones (γ-hemolysis) appearing around the bacterial colonies were considered to indicate that the strains were non-hemolytic.

#### Antimicrobial Resistance

The antimicrobial resistance of the isolated strain was tested by the disk diffusion method (29), with some modifications. Nine antibiotic discs (6 mm, Oxoid, UK) including penicillin G, ampicillin, cefamandole, azithromycin, streptomycin, gentamicin, tetracycline, vancomycin, and chloramphenicol were selected and analyzed according to the recommendations of the European Food Safety Authority for probiotic strains.

Bacterial pellets from overnight MRS broth cultures were harvested (8,000 rpm and 4°C for 10 min), washed twice with PBS and then resuspended in PBS to obtain a bacterial solution in a logarithmic growth cycle (0.5 McFarland suspension). After that, 100 μL aliquots of the bacterial solution were spotted onto MRS agar plates. Finally, the antibiotic discs were applied to the surface of MRS agar plates within 15 min, and the agar plates were transported to a 37°C incubator. After 24 h of incubation, the inhibition zone diameters/mm (IZD) were measured with a Vernier caliper. Since antimicrobial resistance is studied by using breakpoint values to classify microorganisms as resistant or susceptible, neither EUCAST nor CLSI guidelines have been defined for the breakpoints of the disc-diffusion method for studying antibiotic resistance of LAB (30, 31). Therefore, the breakpoints were defined and classified for each antimicrobial based on the CLSI guidelines (Table 1), and the results were compared with the existing literature to verify the classification of resistance (18, 32). Separate experiments were performed in triplicate.

**Table 1:** Antimicrobial resistance of the isolated strains.

**Antimicrobial classes**	**Antimicrobial agents**	**Disk dose (μg)**	**Inhibition zone diameters/mm**			**Antimicrobial**
			**(IZD)** ^**a**^			**susceptibility^**a**^**
			**R**	**I**	**S**	***Z12***
β-lactam antibiotics	Penicillin G	10	≤ 28		≥29	36.33 ± 0.62^S^
	Ampicillin	10	≤ 28		≥29	22.00 ± 0.82^R^
	Cefamandole	30	≤ 14	15~17	≥18	25.40 ± 0.65^S^
Glycopeptides	Vancomycin	30			≥15	X ^R^
Aminoglycoside antibiotics	Streptomycin	10	≤ 11	12~14	≥15	X ^R^
	Gentamicin	10	≤ 12	13~14	≥15	X ^R^
Broad-spectrum antibiotics	Tetracycline	30	≤ 14	15~18	≥19	X ^R^
	Chloramphenicol	30	≤ 12	13~17	≥18	X ^R^
Macrolides	Azithromycin	30	≤ 13	14~17	≥18	15.00 ± 0.82^I^

a *R, Resistant; I, Intermediate; S, Susceptible; X, No inhibition zone observed*.

### *In vivo* Safety Evaluation

#### Animal Experimental Procedure and Sample Preparation

For *in vivo* safety evaluation, 60 4-week-old male Charles River CD-1 (ICR) mice were obtained from Liaoning Changsheng Biotechnology Co., Ltd. All mice were housed at 25 ± 3°C and 55 ± 15% humidity with free access to feed and water. After 1 week of acclimatization, the mice were randomly divided into 4 different groups of 15 animals each. Mice in the control group (CON) were gavaged with 0.2 mL of 0.9% sterile saline, while mice in the other three groups were gavaged with 0.2 mL of *Z12* at doses of 10^7^ (LZ12), 10^8^ (MZ12), and 10^9^ (HZ12) CFU/mL. Body weight and food intake were measured every 3 days. After 21 days of daily gavaging, the mice were fasted for 12 h and then anesthetized with 10% chloral hydrate (0.0350 mL/10 g). Blood was rapidly collected by heart puncture, and serum was harvested by centrifugation (4,000 rpm and 4°C for 10 min) for further analysis. Organs, including the liver, kidney, spleen, and thymus, were collected and weighed after the mice were sacrificed, and organ coefficients were calculated as organ weight/body weight × 100 (33).

#### Mouse Serum Analysis

The aspartate aminotransferase (AST), alanine aminotransferase (ALT), and blood urea nitrogen (BUN) levels as well as the malondialdehyde (MDA) content and superoxide dismutase (SOD) activity in serum were determined by use of commercial ELISA kits (Nanjing Jian Cheng Bioengineering Inc., China). The data were measured by an ELISA plate reader (HKM, Guangdong, China) or biochemical automatic analyzer (Selectra-E, Holland).

### Statistical Analysis

The data are presented as the mean ± standard deviation (SD) and analyzed using *t*-tests and one/two-way analysis of ANOVA (GraphPad Prism software) with the default parameters according to experience. Statistically significant differences between groups were considered at *P* < 0.05.

## Results

### Antimicrobial Activity

**Figure 1:**
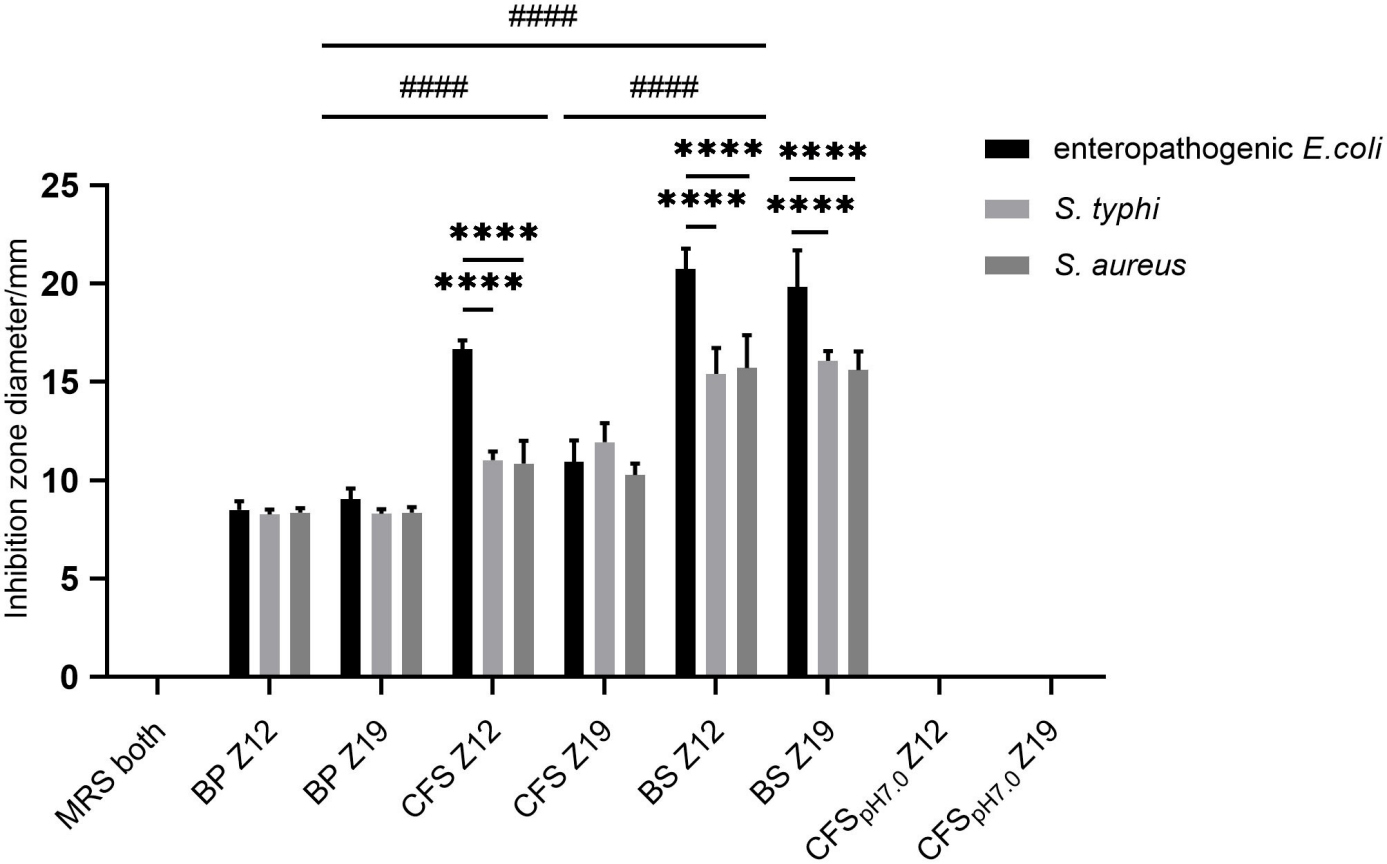
The inhibitory effects of isolated strains against pathogenic indicator bacteria. Values are displayed as the mean ± SD, ****p < 0.0001 indicates differences between two *L. curvatus* strains; ^####^*p* < 0.0001 indicates differences among the BP, CFS, and BS.

Twenty strains (*Z1* to *Z20*) were isolated from rumen fluid (data not shown). Among them, two isolates (*Z12* and *Z19)* exhibited excellent antimicrobial activity against pathogenic indicator bacteria (Figure 1). Therefore, we chose these two isolated strains for further research. The BS and CFS of *Z12* and *Z19* presented a significant inhibitory effect against enteropathogenic *E. coli, S. typhi* and *S. aureus* compared with the BP (*p* < 0.0001), and the inhibitory effect of the BS was markedly higher than that of the CFS (*p* < 0.0001). Moreover, *Z12* and *Z19* significantly inhibited enteropathogenic *E. coli* compared with *S. typhi* and *S. aureus* (*p* < 0.0001), but no significant difference was observed between *S. typhi* and *S. aureus*. In addition, neither MRS broth nor CFS_pH7.0_ had inhibitory effects on pathogens.

### Identification of Isolated Strains

**Figure 2:**
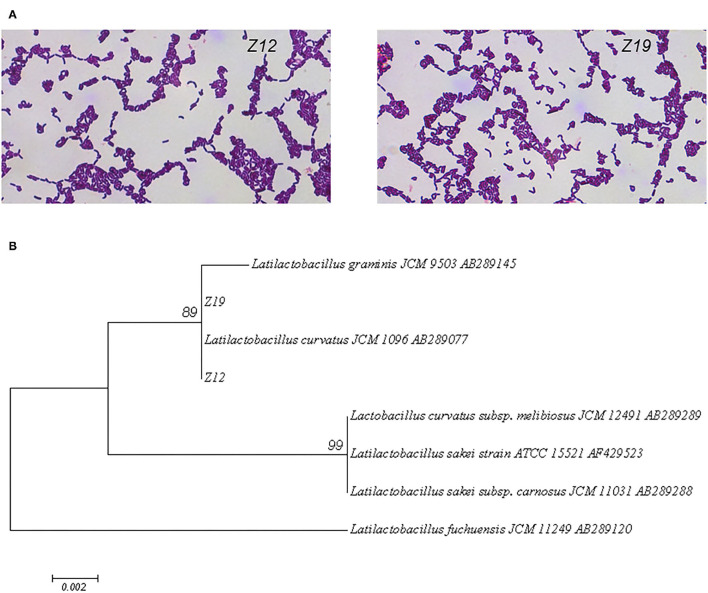
Identification of the isolated strains by morphology and 16S rDNA sequences. **(A)** Gram staining and microscopic examination of isolated strains. **(B)** A phylogenetic tree of 16S rDNA sequences of isolate strains was constructed by MEGA 7.0 software.

On agar plates, the isolated bacterial (*Z12* and *Z19*) colonies were small, round (diameter of 1–2 mm), milky white, and opaque, with neat and smooth edges. Under a light microscope, the isolated strains were gram-positive bean-shaped rods with rounded ends, curved, in pairs or short chains, spore free and flagella free (Figure 2A). Figure 2B shows that the two isolates were identified as *Latilactobacillus curvatus*, named *Latilactobacillus curvatus Z1*2 and *Z19* (97% sequence homology to GenBank sequences).

### Resistance to Artificial Gastrointestinal Models

**Figure 3:**
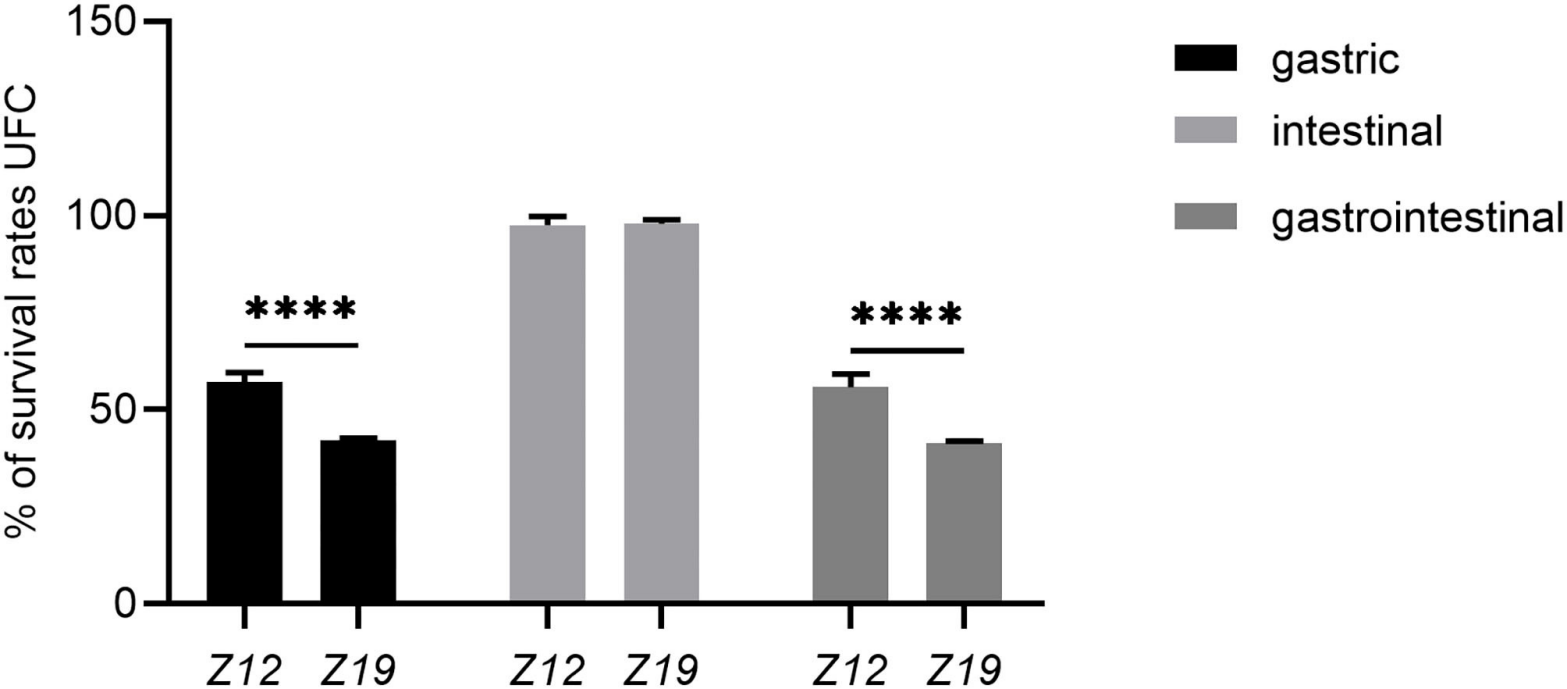
Survival rates of isolated strains after exposure to artificial gastric juice, intestinal fluid, and GIT models. Values were displayed as the mean ± SD, ****p < 0.0001 indicates differences between the two *L. curvatus* strains.

The viability of isolated strains in artificial gastric juice and intestinal fluid was determined by using artificial gastrointestinal models. The survival rates of isolates are shown in Figure 3. The initial surviving cell numbers of *Z12* and *Z19* were 9.52 ± 0.18 and 9.46 ± 0.10 (log CFU/mL), which were reduced to 5.44 ± 0.14 and 4.00 ± 0.02 (log CFU/mL) after exposure to artificial gastric juice. Thus, the survival rates of *Z12* and *Z19* were 57.20% and 42.24% (*p* < 0.0001). Moreover, the surviving cell numbers of *Z12* and *Z19* after treatment with artificial intestinal fluid remained at 9.28 ± 0.11 and 9.26 ± 0.14 (log CFU/mL), and the survival rates were 97.61 and 97.91%. Overall, the survival rates of *Z12* and *Z19* after gastrointestinal model simulation were 55.87 and 41.36%, respectively, and *Z12* showed significantly higher resistance to artificial gastrointestinal models than *Z19* (*p* < 0.0001).

### Cell Surface Hydrophobicity and Autoaggregation Ability

**Figure 4:**
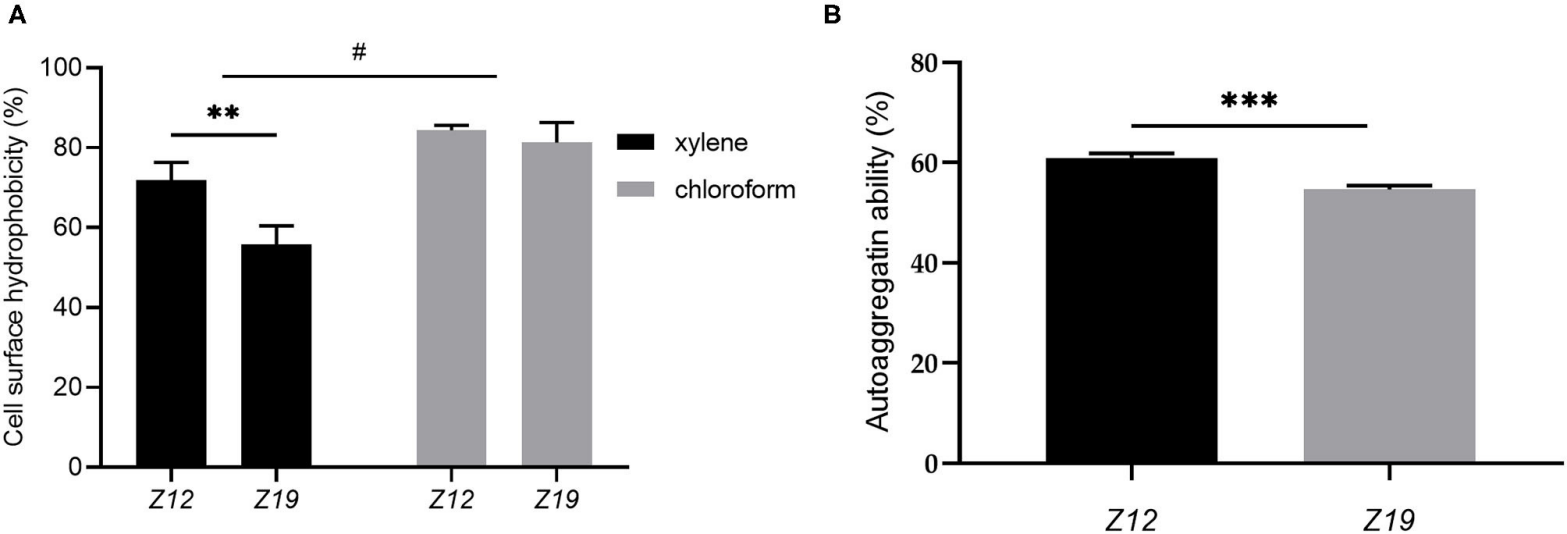
CSH and autoaggregation ability of *L. curvatus* strains. **(A)** CSH based on partitioning to xylene and chloroform and **(B)** autoaggregation ability of *L. curvatus* strains. Values displayed are the mean ± SD, **p < 0.01 and ***p < 0.001 indicate differences between the two *L. curvatus* strains; ^#^*p* < 0.05 indicates differences between xylene and chloroform.

The isolated *L. curvatus* strains showed suitable CSH and autoaggregation ability (Figure 4). The CSH of *Z12* and *Z19* partitioning to xylene were 71.95 ± 3.56% and 55.74 ± 3.85% (*p* < 0.01), while that for partitioning to chloroform was 81.35 ± 4.05% and 84.41 ± 0.99%, respectively (Figure 4A). The autoaggregation abilities of *Z12* and *Z19* reached 60.98 ± 0.72% and 54.64 ± 0.67%, respectively (*p* < 0.001; Figure 4B).

### Safety Evaluation

#### Hemolytic Activity and Antimicrobial Resistance

According to the above results, *Z12* showed better probiotic characteristics than *Z19*. Therefore, we evaluated only the safety of *Z12* in the following study. *Z12* was negative for hemolytic activity (data not shown), and its antimicrobial resistance levels are presented in Table 1. *Z12* was highly susceptible to penicillin G and cefamandole, showed intermediate resistance to azithromycin and showed resistance to ampicillin, vancomycin, streptomycin, gentamicin, tetracycline, and chloramphenicol.

#### Effect of Z12 on Body Weight, Food Intake, and Organ Coefficients in Mice

**Figure 5:**
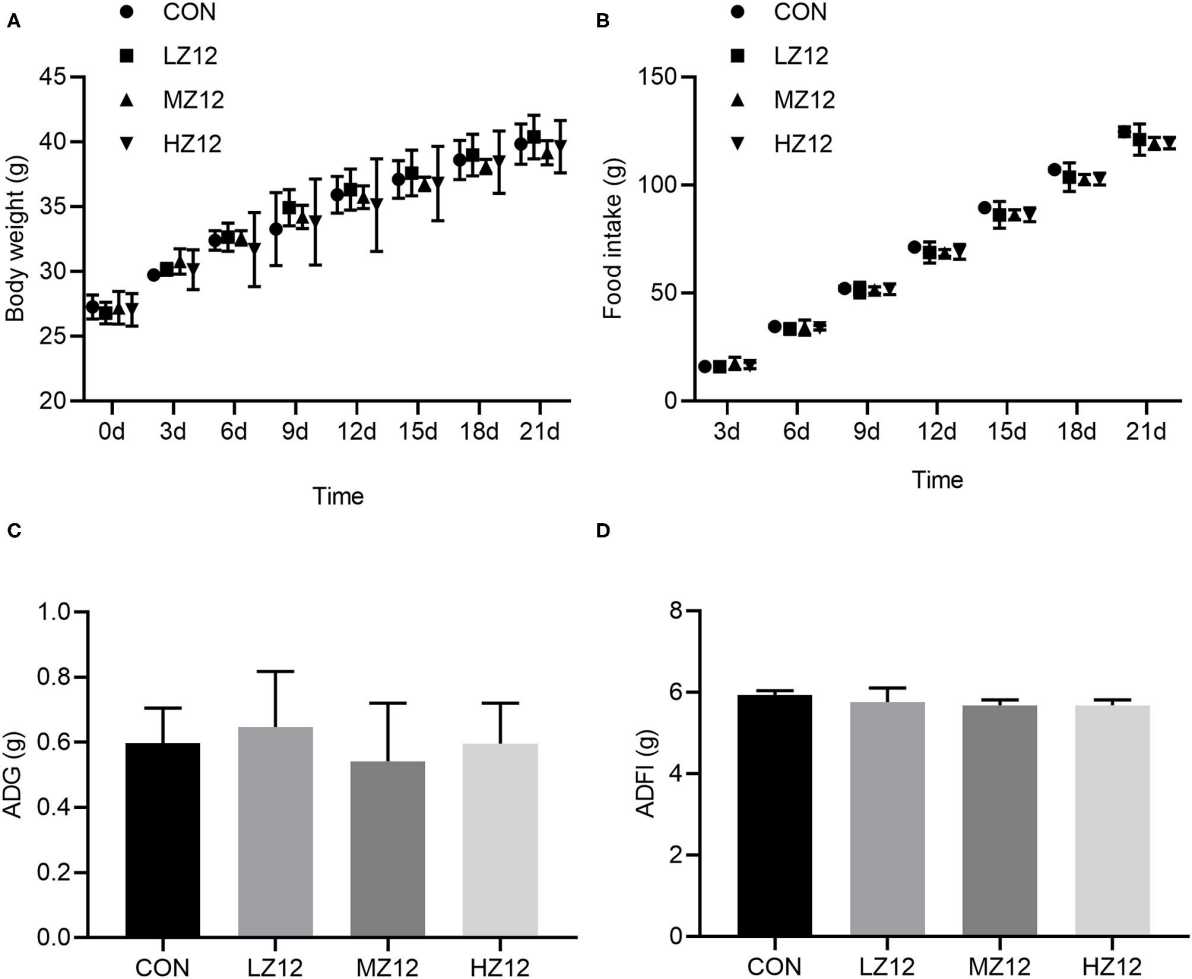
Effect of LZ12 supplementation on body weight and food intake in mice. Body weight **(A)** and food intake **(B)** were measured every 3 days, and ADG **(C)**, and ADFI **(D)** were calculated at the end of the trial period. Values were displayed as the mean ± SD.

**Figure 6:**
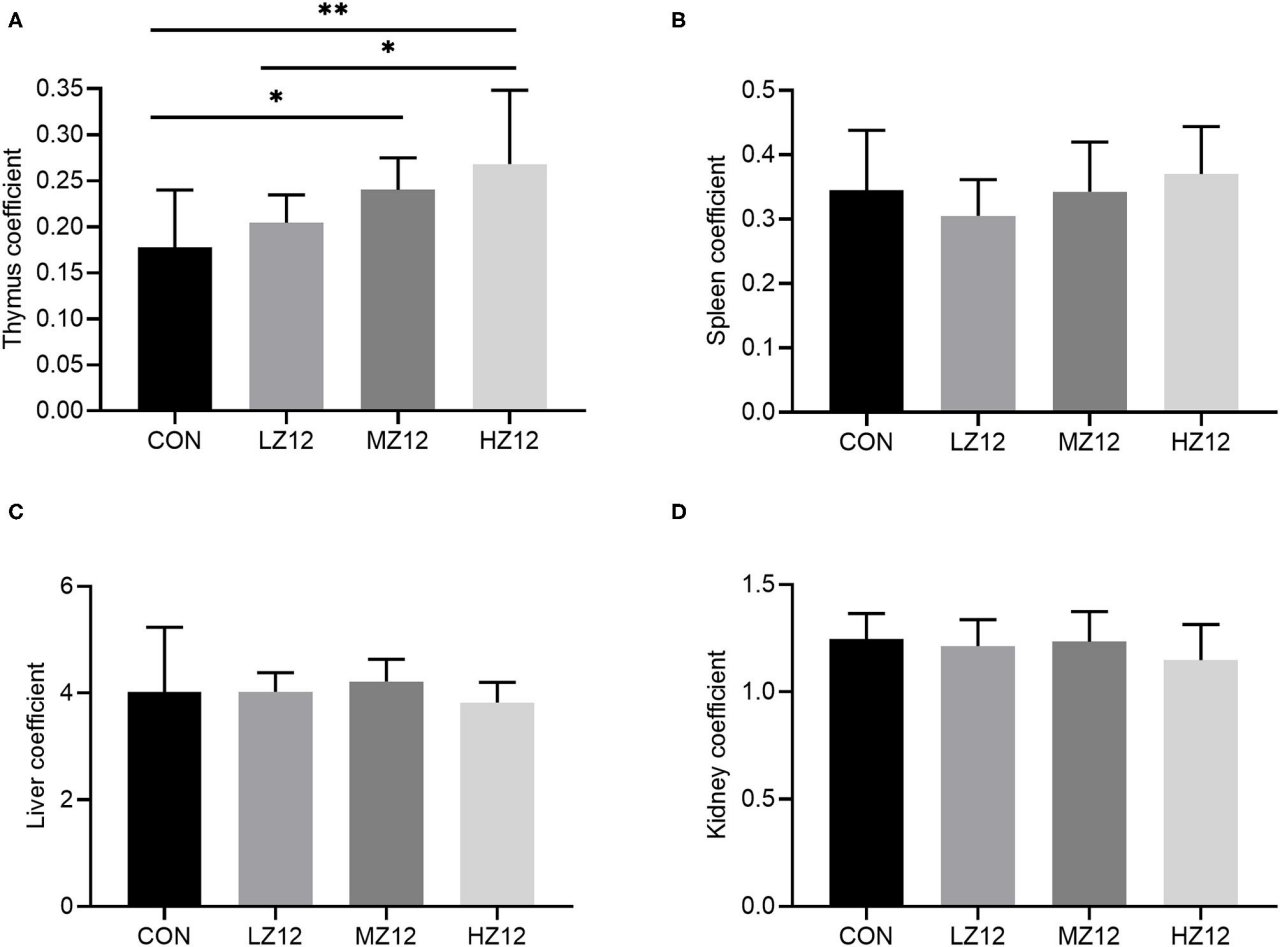
Effect of LZ12 supplementation on organ coefficients in mice. The thymus coefficient **(A)**, spleen coefficient **(B)**, liver coefficient **(C)**, and kidney coefficient **(D)** were calculated as thymus/spleen/liver/kidney weigh/body weigh × 100. Values displayed are the mean ± SD, *p < 0.05 and **p < 0.01 indicate differences among the four different groups.

Body weight, food intake and organ coefficients are commonly used health status parameters to evaluate the safety of isolates. First, we studied the influence of *Z12* supplementation on body weight and food intake in mice. The results showed that supplementation with different doses of *Z12* had no marked effect on body weight, food intake, average daily gain (ADG) or average daily food intake (ADFI) compared with those of the CON group (Figures 5A–D). Next, we investigated the impact of *Z12* on organ coefficients. As shown in Figure 6, the HZ12 and MZ12 groups had significantly higher thymus coefficients than the CON group; moreover, the thymus coefficient in the HZ12 group was markedly higher than that in the LZ12 group (Figure 6A), indicating that the addition of 10^8^ and 10^9^ CFU/mL *Z1*2 can promote the development of the thymus, a major immune organ. The spleen, liver and kidney coefficients showed no significant effect among all groups (Figures 6B–D).

#### Effect of Z12 on Serum Biochemical Parameters and Antioxidant Indexes in Mice

**Figure 7:**
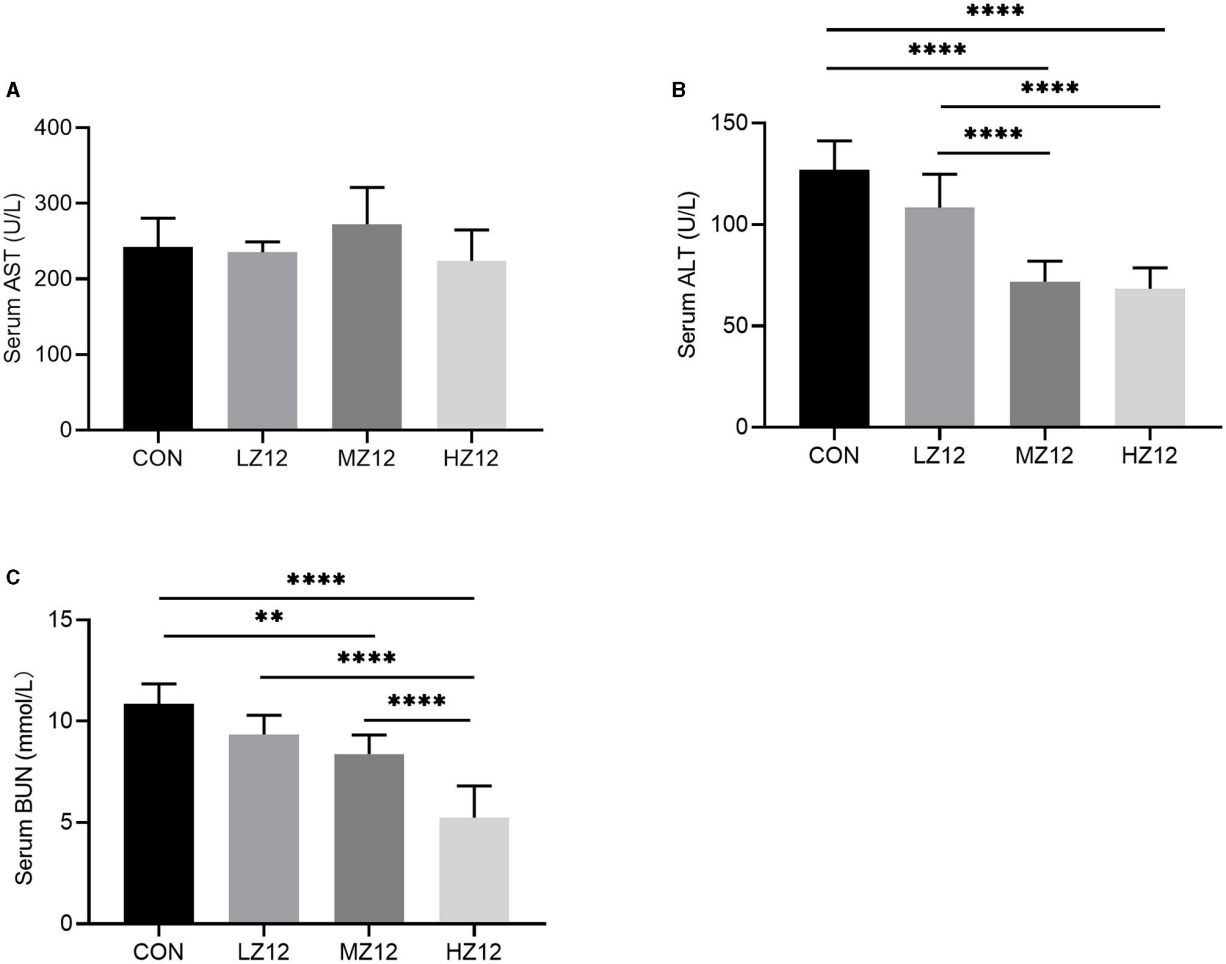
Effect of Z12 on serum biochemical parameters in mice. The levels of serum AST **(A)**, ALT **(B)**, and BUN **(C)** were determined by use of commercial ELISA kits. Values are displayed as the mean ± SD, **p < 0.01 and ****p < 0.0001 indicate differences among the four different groups.

**Figure 8:**
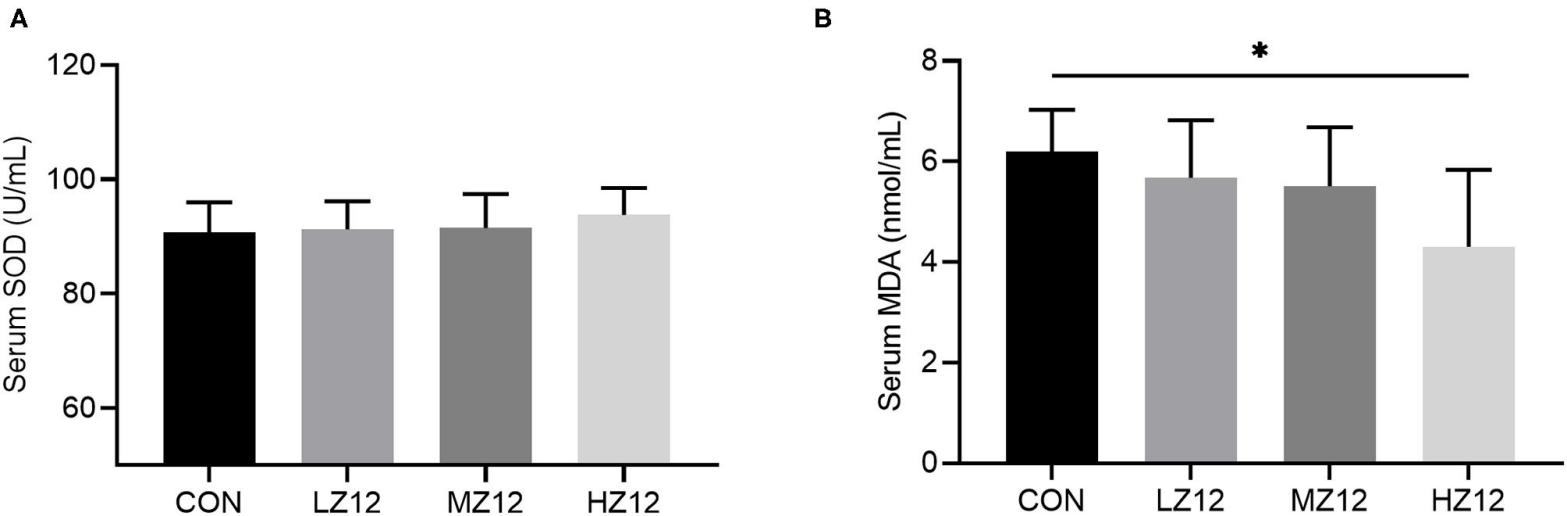
Effect of LZ12 supplementation on antioxidant indexes in mice. The levels of serum SOD **(A)** and MDA **(B)** were determined by use of commercial ELISA kits. Values are displayed as the mean ± SD, *p < 0.05 indicates differences among the four different groups.

The mice in the HZ12 and MZ12 groups had significantly decreased serum ALT and BUN levels compared with those of the mice in the LZ12 and CON groups (Figures 7B,C). Moreover, the mice in the HZ12 group showed a significant decrease in the serum MDA content compared with that of the mice in the CON group (Figure 8B); however, the serum AST levels and SOD activity showed no marked effects in the different groups, but with the increase in *Z12* dose, serum SOD activity showed an increasing trend (Figure 7A, Figure 8A).

## Discussion

Probiotics are non-pathogenic living microorganisms that may have beneficial effects on the host by increasing the number of intestinal microorganisms, thereby inhibiting the growth of pathogens (18). *L. curvatus* is a member of the genus *Latilactobacillus*, which has excellent health benefits. In 2013, it was listed as a qualified presumption of safety (QPS)-recommended biological agent added to food or feed by the European Food Safety Administration (34). Earlier studies have reported that *L. curvatu*s species were mainly isolated from some fermented meat, plants, and dairy products, as well as the gut of monogastric animals (35, 36). However, no related study has been conducted in ruminants. In the present study, we identified two strains of *L. curvatu*s from fresh rumen fluid of an adult female domestic sika deer and evaluated their probiotic and safety characteristics, providing a theoretical basis for the study of sika deer-derived probiotics as feed additives against gastrointestinal inflammation in sika deer.

In recent years, there has been increasing interest in the isolation of new strains that produce antimicrobial substances because of the potential industrial application of probiotics for inhibiting the growth of pathogens. In this study, *Z12* and *Z19* exhibited strong antimicrobial activity against indicator pathogens, including enteropathogenic *E. coli* (ATCC 25922), *S. typhi* (ATCC14028), and *S. aureus* (ATCC25923). Moreover, the antimicrobial activity of BS and CFS was stronger than that of BP. These results suggested that *Z12* and *Z19* have excellent antimicrobial activity and that their antimicrobial activity might be mediated by their metabolites. An earlier study reported that LAB can produce a variety of antibacterial metabolites, including organic acids, inhibitory enzymes, hydrogen peroxide, and bacteriocins (16). In the present study, after the pH of the CFS was neutralized (CFS_pH7.0_), their inhibitory effects on pathogens completely disappeared, indicating that the inhibitory effects of *L. curvatus* may be due to the produced organic acids.

Exposure to the GIT is the main stress that could reduce the viability of most ingested probiotics due to the low pH (1.5–3.5) value of gastric juice and the antimicrobial activity of bile salts and pancreatic juice in intestinal fluid (37). Hence, probiotics must be able to survive in gastrointestinal conditions to perform their physiological functions. Previous studies have shown that *L*. c*urvatus PA40* and *L*. c*urvatus DN317* isolated from Kimchi (a traditional fermented Korean side dish) and chicken ceca have high survival rates under simulated gastric juice (pH 2.5) and bile acid conditions (18, 36). Similar results were also found for *Z12* and *Z19*, which remained viable after artificial gastric juice (pH 2.0, survival rates of 57.14 and 42.28%, respectively) and intestinal fluid (survival rates of 97.48 and 97.89%, respectively) conditions. These results indicate that strains *Z12* and *Z19* could survive and perform their physiological functions under gastrointestinal conditions but *Z12* has a higher tolerance than *Z19*.

In addition, measuring the CSH and autoaggregation ability of probiotics are generally considered to be suitable methods for identifying potential adherent bacteria, as they are associated with adhesion to epithelial cells and play a significant role in preventing pathogen colonization (26). CSH >50% is considered to be highly hydrophobic (38). In this work, the hydrophobicity of the two isolates ranged from 55.74 to 84.41% using xylene and chloroform; thus, the two isolates presented strong affinities to the tested solvents. In addition, *Z12* also showed high autoaggregation ability (60.98%), which was consistent with results for *L. curvatus* strains A61 and DN317 reported in previous studies (36, 39). However, the autoaggregation ability of *Z19* (54.64%) was significantly lower than that of *Z12*, which may be due to the complex interactions among molecules on the surface of the bacteria, such as secretory factors and proteins. It has been shown that internal factors may determine the potential aggregation phenotype (17). Overall, *Z12* displayed more suitable CSH and autoaggregation abilities, which could lead to colonization of the GIT and more effectively prevention of pathogenic bacteria than that by *Z19*.

Notably, LAB as probiotics must be safe for the host and have no hemolytic activity (17) or transferable and acquired antimicrobial resistance (36). In this study, *Z12* had no lytic activity against sheep blood erythrocytes, so it can be considered a safe candidate for probiotics. Generally, LAB are sensitive to broad-spectrum antibiotics (e.g., tetracycline and chloramphenicol), macrolides (azithromycin), glycopeptides (vancomycin), and β-lactam antibiotics (e.g., ampicillin, penicillin, and cephalothin) but resistant to aminoglycosides (e.g., streptomycin and gentamicin) (25, 32). However, in our study, some contradictory resistance results were found for *Z12*, which displayed resistance to ampicillin, vancomycin, tetracycline, and chloramphenicol but sensitivity to penicillin G and cefamandole and intermediate resistance to azithromycin. The differences in antimicrobial resistance and susceptibility to antimicrobials between LAB may be due to the impermeability of the cell wall (40) or multidrug transporters and defects in cell wall autolysis systems (41). Furthermore, *L. curvatus DN317* has been found to be sensitive to ampicillin, vancomycin, gentamicin and streptomycin (36) and resistant to chloramphenicol. *L. curvatus PA40* has been found to be moderately resistant to tetracycline, vancomycin and erythromycin (18), while *L. curvatus A61* (39) has been found to be resistant to ampicillin. These results suggest that there are also resistance differences even between *L. curvatus* strains, which possibly varies depending on the resistance genes of the subspecies. To date, however, no data have been published on antimicrobial resistance genes of *L. curvatus*. In fact, there are various opinions as to whether probiotics showing resistance to specific antimicrobials are desirable since antimicrobial-resistant probiotics also have potentially negative effects, such as the transfer of resistance to intestinal pathogens or antibiotic-induced diarrhea (25). However, previous studies have shown that mobile elements such as phage, transposases and insertion sequences were not found in the complete genomes of *L. curvatus* SRCM103465 (CP035110.1), *L. curvatus* MARS6 (CP022474), and *L. curvatus* 20,019 (CP026116.1), as well as their upstream and downstream 5 kbp sequences downloaded from NCBI, indicating a low transfer risk of antimicrobial resistance genes in *L. curvatus* species (32).

Although *L. curvatus* was listed as a QPS-recommended biological agent added to feed, potential toxicity-related factors must be considered and evaluated. The impacts on animal health are usually used to assess the safety of probiotic isolates. In the current study, no noteworthy differences were observed in mouse body weight or food intake among the four disparate groups. The thymus coefficient, a marker of immune system development (42, 43), was increased markedly in the MZ12 and HZ12 groups. Moreover, when the body is subjected to stimuli, induced oxidative stress can cause damage to some organs, such as the liver and kidney (44). In our study, mice were gavaged daily for 21 days with different doses of *Z12*, which may result in certain stimulation or toxicity to mice. Therefore, some major indexes suggestive of hepatotoxicity (AST and ALT) and nephrotoxicity (BUN) as well as indexes of oxidative injury (MDA) and antioxidant capacity (SOD) were also examined. The serum levels of ALT and BUN were significantly decreased in the MZ12 and HZ12 groups compared with those in the CON and LZ12 groups; furthermore, the MDA content decreased markedly in the HZ12 group, while SOD activity, although there was no significant difference among all the groups, showed an increasing trend with increasing *Z12* dose. These results indicated that high-dose *Z12* supplementation not only has no toxic effects on mice but also plays an important role in promoting the development of the immune system and alleviating organ damage caused by oxidative stress. *Z12* isolated from rumen fluid is safe and has probiotic characteristics, including antimicrobial activity, tolerance to artificial gastrointestinal models, CSH and autoaggregation ability. In addition, high-dose (10^9^ CFU/mL) supplementation with *Z12* has an excellent ability to promote immune system development and alleviate the damage to organs caused by oxidative stress. Hence, *Z12* could be used as a potential sika deer-derived probiotic but should not be administered in combination with therapeutic antimicrobials, such as penicillin G and cefamandole.

## Data Availability Statement

The datasets generated for this study are deposited in the NCBI sequence read archive, and the accession numbers are OK206086-OK206087.

## Ethics Statement

The animal study was reviewed and approved by The Institute of Special Animal and Plant Sciences, Chinese Academy of Agricultural Sciences Institutional Animal Care and Use Committees (No. ISAPSAEC-2021-33). Written informed consent was obtained from the owners for the participation of their animals in this study.

## Author Contributions

GL and SC: study design, editing, and funding acquisition. YZ: investigation, analysis, original draft, and editing. SL, MZ, and XY: investigation writing-review and editing. All authors read and approved the final manuscript.

## Funding

The writing of the manuscript was supported by the Ministry of Science and Technology of the People's Republic of China (Key Technologies Research and Development Program) under the grant code 2018YFC1706600 and the Start-up Fund for Scientific Research of High-level Talents of Qingdao Agricultural University (1121009).

## Conflict of Interest

The authors declare that the research was conducted in the absence of any commercial or financial relationships that could be construed as a potential conflict of interest.

## Publisher's Note

All claims expressed in this article are solely those of the authors and do not necessarily represent those of their affiliated organizations, or those of the publisher, the editors and the reviewers. Any product that may be evaluated in this article, or claim that may be made by its manufacturer, is not guaranteed or endorsed by the publisher.
